# Predictors of Burden for First-Ever Stroke Survivor’s Long-Term Caregivers: A Study of KOSCO

**DOI:** 10.3390/medicina60040559

**Published:** 2024-03-29

**Authors:** Jin-Won Lee, Min Kyun Sohn, Jongmin Lee, Deog Young Kim, Yong-Il Shin, Gyung-Jae Oh, Yang-Soo Lee, Min Cheol Joo, So Young Lee, Junhee Han, Jeonghoon Ahn, Yun-Hee Kim, Min-Keun Song, Won Hyuk Chang

**Affiliations:** 1 Department of Physical and Rehabilitation Medicine, Chonnam National University Medical School, Gwangju 61469, Republic of Korea; 2 Department of Rehabilitation Medicine, College of Medicine, Chungnam National University, Daejeon 35015, Republic of Korea; 3 Department of Rehabilitation Medicine, Konkuk University School of Medicine, Seoul 05029, Republic of Korea; 4 Department and Research Institute of Rehabilitation Medicine, Yonsei University College of Medicine, Seoul 03722, Republic of Korea; 5 Department of Rehabilitation Medicine, Pusan National University School of Medicine, Pusan National University Yangsan Hospital, Yangsan 50612, Republic of Korea; 6 Department of Preventive Medicine, Wonkwang University School of Medicine, Iksan 54538, Republic of Korea; 7 Department of Rehabilitation Medicine, Kyungpook National University School of Medicine, Kyungpook National University Hospital, Daegu 41944, Republic of Korea; 8 Department of Rehabilitation Medicine, Wonkwang University School of Medicine, Iksan 54538, Republic of Korea; 9 Department of Rehabilitation Medicine, Jeju National University Hospital, Jeju National University School of Medicine, Jeju City 63243, Republic of Korea; 10 Department of Statistics, Hallym University, Chuncheon 24252, Republic of Korea; 11 Department of Health Convergence, Ewha Womans University, Seoul 03760, Republic of Korea; 12 Department of Physical and Rehabilitation Medicine, Center for Prevention and Rehabilitation, Heart Vascular Stroke Institute, Samsung Medical Center, Sungkyunkwan University School of Medicine, Seoul 06355, Republic of Korea

**Keywords:** caregiver burden, predictors, long-term care, first-ever stroke, stroke rehabilitation

## Abstract

Long-term changes in caregiver burden should be clarified considering that extended post-stroke disability can increase caregiver stress. We assessed long-term changes in caregiver burden severity and its predictors. This study was a retrospective analysis of the Korean Stroke Cohort for Functioning and Rehabilitation. Patients with an acute first-ever stroke were enrolled from August 2012 to May 2015. Data were collected at 6 months and 6 years after stroke onset. The caregiver burden was measured with a subjective caregiver burden questionnaire based on the Korean version of the Caregiver Burden Inventory. The caregivers’ characteristics and patients’ clinical and functional status were also examined at each follow-up. A high caregiver burden, which suggests a risk of burnout, was reported by 37.9% and 51.7% of caregivers at 6 months and 6 years post-stroke, respectively. Both the caregiver burden total score and proportion of caregivers at risk of burnout did not decrease between 6 months and 6 years. The patients’ disability (OR = 11.60; 95% CI 1.58–85.08; *p* = 0.016), caregivers’ self-rated stress (OR = 0.03; 95% CI 0.00–0.47; *p* = 0.013), and caregivers’ quality of life (OR = 0.76; 95% CI 0.59–0.99; *p* = 0.042) were burden predictors at 6 months. At 6 years, only the patients’ disability (OR = 5.88; 95% CI 2.19–15.82; *p* < 0.001) and caregivers’ psychosocial stress (OR = 1.26; 95% CI 1.10–1.44; *p* = 0.001) showed significance. Nearly half of the caregivers were at risk of burnout, which lasted for 6 years after stroke onset. The patients’ disability and caregivers’ stress were burden predictors in both subacute and chronic phases of stroke. The findings suggest that consistent interventions, such as emotional support or counseling on stress relief strategies for caregivers of stroke survivors, may reduce caregiver burden. Further research is needed to establish specific strategies appropriate for Korean caregivers to alleviate their burden in caring for stroke patients.

## 1. Introduction

Stroke is a leading cause of disability worldwide [1]. The 2019 Global Burden of Disease study named stroke as the second-leading cause of death and the third-leading cause of death and disability combined [1]. From 1999 to 2019, the absolute stroke incidence rate increased by 70.0%, and disability-adjusted life years due to stroke increased by 32.0% [1]. The number of stroke survivors may increase from 3,718,785 in 2015 to 4,631,050 in 2035 [2]. Fortunately, stroke mortality has decreased worldwide [3,4]. The referral of inpatients with acute stroke to rehabilitation medicine specialists could decrease stroke-related mortality [5].

However, post-stroke disability remains a significant socioeconomic burden on patients, caregivers, and society, because many stroke survivors have some degree of sequelae [6]. Long-term survivors of stroke with severe disability may stay in the rehabilitation unit and experience repeated admissions to various hospitals [7]. The disease burden of stroke is accompanied by a substantial economic burden; the total (direct and indirect) costs of stroke have been estimated to be USD 40.1 billion annually in the United States (US) [8] and KRW 4135.78 billion in 2017 in South Korea [9].

Moreover, stroke has a prolonged physical and emotional impact on caregivers such as family and friends. More than 48% of caregivers of patients with stroke reported health problems, and two-thirds have some difficulties in their social activities [10,11]. Caregiver burden is a subjective perception of a caregiver’s workload. Previous studies have identified specific patient variables (e.g., disability, activities of daily living, anxiety, and depression) and caregiver variables (e.g., caregiver workload, anxiety, and depression) as burden predictors [12,13,14,15,16,17,18,19,20,21,22]. Several studies revealed that caregiver burden can decrease over time [14,15,23,24] by adapting to lifestyle changes and developing coping strategies [25]. Conversely, other studies indicated that caregivers experience persistent fatigue and anxiety [26], and that caregiver burden and quality of life are maintained over time [26,27,28]. However, most of these studies only analyzed cases within 1 year post-event.

Among studies with a longer duration, one study reported that caregiver burden was decreased within 3 years [24], and another study observed a gradual decrease from 6 months to 5 years post-event [23]. In the present study, we investigated the burden perceived by the caregivers of stroke survivors for up to 6 years post-event through a follow-up questionnaire. The data obtained from assessing the changes in the caregiver burden and its predictors over time could contribute to the development of time-specific interventions for alleviating caregiver burden.

## 2. Materials and Methods

### 2.1. Study Design

This was an analysis of the Korean Stroke Cohort for Functioning and Rehabilitation (KOSCO), which is a prospective multicenter cohort of stroke survivors admitted to 9 tertiary university hospitals representing each district in South Korea, with a 10-year follow-up period. We enrolled patients from August 2012 to May 2015. Inclusion criteria were as follows: (1) first-ever acute stroke (ischemic or hemorrhagic) diagnosed through computerized tomography or magnetic resonance imaging; (2) age ≥ 19 years at stroke onset; and (3) within 7 days from symptom onset before inclusion. Exclusion criteria were as follows: (1) transient ischemic attack; (2) stroke history; (3) traumatic intracerebral hemorrhage; and (4) not Korean [29]. Detailed information on the settings and protocol of the KOSCO study has been presented in a previous publication [29].

### 2.2. Study Population and Procedures

**Figure 1:**
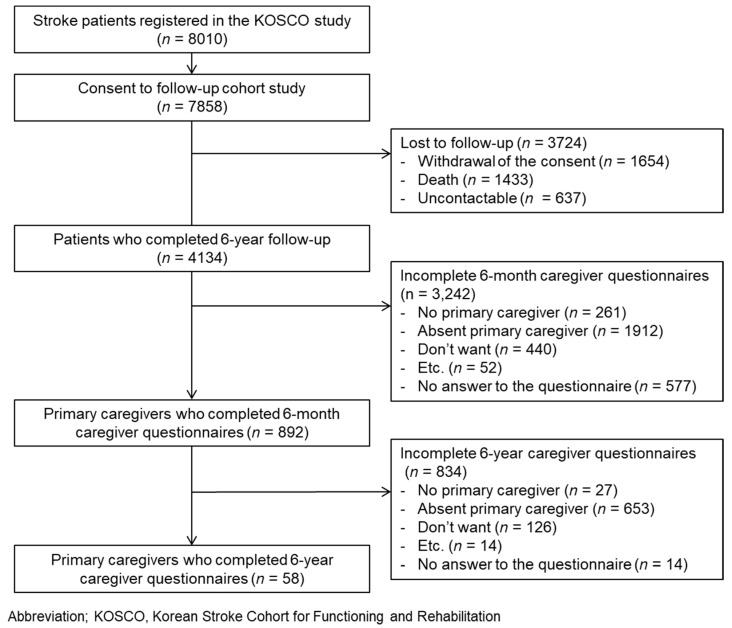
Flowchart of patient and caregiver inclusion.

Of the 8010 patients enrolled in the KOSCO study, 7858 of them agreed to a long-term follow-up. Among the 7858 patients, 4134 patients who completed face-to-face interviews at 6 years post-stroke were screened. The follow-up time points were at discharge and 7 days, 3 months, 6 months, 12 months, 18 months, 24 months, 36 months, 48 months, 60 months, and 72 months from onset of stroke. Among the primary caregivers, 892 caregivers responded to the survey at 6 months post-stroke, and 58 of them also responded at 6 years post-event (Figure 1). Using the data from the final 58 patient/caregiver pairs, changes in caregiver burden and its predictors over time were analyzed.

### 2.3. Demographic Data and Measurement Tools

The following demographic data were collected at the time of admission: age, sex, body mass index (BMI), and type of stroke. Furthermore, we used the following measurement tools: Caregiver Burden Inventory (CBI) [30], National Institute of Health Stroke Scale (NIHSS) [31], and Charlson Comorbidity Index (CCI) [32], including Combined Condition and Age-related Score (CCAS), also known as Age-adjusted CCI. The modified Rankin Scale (mRS) [33], Korean Mini Mental State Examination (K-MMSE) [34], Fugl-Meyer Assessment (FMA) [35], Korean Version of the Frenchay Aphasia Screening Test (K-FAST) [36], Functional Ambulatory Category (FAC) [37], and American Speech-language Hearing Association National Outcome Measurement System Swallowing Scale (ASHA-NOMS) [38] were also used for specific variables. Evaluation with these tools was performed on the 7th day post-stroke and at each follow-up. Furthermore, the Korean Modified Barthel Index (K-MBI) [39], Geriatric Depression Scale-Short Form (GDS-SF) [40], and Euro Quality of Life-5 Dimension (EQ-5D) [41] were used to evaluate patient and caregiver characteristics. The Psychosocial Wellbeing Index-Short Form (PWI-SF) [42] was used for caregiver assessment. Data were collected at discharge and each follow-up.

### 2.4. Factors Related to Caregiver Burden

- Dependent factors related to caregiver burden

Caregiver burden was measured using a subjective caregiver burden questionnaire. This questionnaire was based on the Korean version of the CBI with proven reliability and validity [43]. The five CBI subscales include time-dependence, developmental, physical, social, and emotional burdens. Several items were selected for each subscale (time-dependence (2), developmental (3), physical (2), social (4), and emotional (2); a total of 13 items), and there were 2 additional items related to financial burden, resulting in a total of 15 items. Each item was rated on a 1 to 5 scale with a total score in the range of 15–75; a higher score indicated higher caregiver burden. The cut-off score was calculated based on a well-known reference score, i.e., a total score > 36 [30], which indicates a risk of “burnout” in CBI (CBI total score, 0–100). Accordingly, the calculated score of 36.6 was used for discretizing the continuous variable, the subjective caregiver burden questionnaire score. Moreover, the means of each of the six subscales were calculated for comparison according to the burden domain.

B. Independent factors related to caregiver burden

  1. Patient’s factors related to caregiver burden variables

Stroke onset was evaluated with the score of the NIHSS [31]. Comorbidities were evaluated with the CCI, which includes the CCAS and Weighted Index of Comorbidity (WIC) [32]; the CCAS refers to the age-adjusted CCI, whereas the WIC refers to the CCI without adjusting for age. The mRS was used to determine the pre-stroke and post-stroke functional status [33]. Functional assessment was also performed using the K-MMSE for cognitive function [34] and the FMA for motor function [35]. Additionally, we used the following measurement tools to evaluate specific functions: K-FAST for language [36], FAC for mobility function [37], and ASHA-NOMS for swallowing function [38]. These tests were conducted on the 7th day post-event and at each follow-up. Activities of daily living were assessed with the K-MBI [39]. Depressive symptoms were evaluated using the GDS-SF [40]. Lastly, health-related quality of life was assessed using the EQ-5D [41]. It is composed of 5 questionnaires: mobility, self-care, usual activities, pain/discomfort, and anxiety/depression. Each question has three response levels. The total score was calculated using the standard weight model determined by the Korea Center for Disease Control and Prevention [44]. The EQ-5D value was obtained by multiplying the total score by 100 was used.

  2. Caregiver’s factors related to caregiver burden

We examined variables that may function as potential predictors using the caregiver survey. Caregiver age (grouped into decades), gender, employment, education level, relationship with patient, relationship level, cohabitation with patient, home care, long-term care institution consignment, hospitalized patient care, social support service use such as home visit services or daycare center, alternative caregiver presence, self-rated health, self-rated stress, and stroke knowledge (motor, language, visual, ataxic symptoms, and acute stage treatment) were recorded. Psychosocial stress was measured with the PWI-SF [42]. This questionnaire consists of 18 questions and screens for common psychiatric disorders such as depression and anxiety. Each question was scored from 0 to 3, and the total score ranged from 0 to 54, with a higher score indicating higher stress. Quality of life was assessed using the EQ-5D, similar to the survey for patients.

### 2.5. Statistical Analysis

We recruited all KOSCO study patients who met the criteria to reach the target number of participants. Experts indicated that sufficient power could be achieved with a standard cohort of 10,636 individuals. A power of more than 90% would be required to address the primary endpoint.

Continuous variables related to patient and caregiver characteristics are presented as means and standard deviations or medians and interquartile intervals, whereas categorical variables are presented as numbers and percentages. Variables with a missing value (patient variables: K-FAST and GDS-SF; caregiver variable: education) were excluded from subsequent data analysis. To investigate changes in patient and caregiver characteristics over time, paired *t*-test for continuous data, McNemar’s test for nominal data, and Wilcoxon test for ordinal data were performed.

The association of each risk factor with caregiver burden was assessed by bivariate analysis using *t*-test or $\chi^{2}$ test. Following bivariate analysis, multivariable binary logistic regression analysis with forward stepwise variable selection was performed to identify independent caregiver burden predictors. The statistical significance level was set at a *p* value of <0.05. Analysis was conducted using SPSS statistical software version 29.0 (SPSS Inc., Chicago, IL, USA).

### 2.6. Data Availability

The data associated with the paper are not publicly available but are available from the corresponding author on reasonable request.

## 3. Results

### 3.1. Comparison of the Included and Excluded Groups

The 58 patient/caregiver pairs who responded to the survey at 6 years post-event were compared with the 834 patient/caregiver pairs who did not respond, which demonstrated that there were no significant differences in the patients’ gender and all caregiver characteristics (Table 1). However, the patients’ age (responders mean [SD], 7.45 [1.14] decades; non-responders mean [SD], 6.93 [1.21] decades, *p* < 0.001), patients’ premorbid mRS (responders median [IQR], 0.0 [0.0–0.0]; non-responders median [IQR], 0.0 [0.0–1.0], *p* = 0.014), initial NIHSS (responders mean [SD], 7.34 [9.10]; non-responders mean [SD], 4.72 [6.45], *p* = 0.035), K-MMSE (responders mean [SD], 17.40 [10.02]; non-responders mean [SD], 24.16 [7.01], *p* < 0.001), FMA (responders mean [SD], 78.84 [30.26]; non-responders mean [SD], 87.15 [25.16], *p* = 0.045), FAC (responders median [IQR], 4.0 [3.0–5.0]; non-responders median [IQR], 5.0 [4.0–5.0], *p* = 0.002), K-FAST (responders mean [SD], 16.45 [9.39]; non-responders mean [SD], 21.56 [8.73], *p* < 0.001), mRS (responders median [IQR], 2.0 [1.0–3.0]; non-responders median [IQR], 1.0 [1.0–2.0], *p* < 0.001), K-MBI (responders mean [SD], 80.52 [28.28]; non-responders mean [SD], 88.76 [21.54], *p* = 0.033), GDS-SF (responders mean [SD], 6.97 [3.93]; non-responders mean [SD], 5.53 [4.03], *p* = 0.029), and EQ-5D (responders mean [SD], 52.81 [39.87]; non-responders mean [SD], 72.20 [30.83], *p* < 0.001) showed significant differences, indicating the better functional and emotional status of non-responders.

### 3.2. Patient and Caregiver Characteristics

A total of 58 caregivers (6 months, mean [SD] age, 6.02 [1.17] decades, 20 males [34.5%]; 6 years, mean [SD] age, 6.64 [1.21] decades; 17 males [29.3%]) completed the caregiver survey both at 6 months and 6 years post-event. Patient and caregiver characteristics are presented in Table 2 and Table 3, respectively. From 6 months to 6 years post-event, the patients’ cognitive function (K-MMSE mean [SD], 17.40 [10.02] to 13.62 [11.91], *p* = 0.002) and activities of daily life (K-MBI mean [SD], 80.52 [28.28] to 72.00 [30.94], *p* = 0.007) were decreased over time. However, there were no changes in the patients’ motor, mobility, swallowing function, and quality of life.

Caregivers were mainly spouses (6 months, 48.3%; 6 years, 46.6%) or children (6 months, 43.1%; 6 years, 44.8%) of patients, and cared for the patients at home (6 months, 75.9%; 6 years, 75.9%). More than half of the caregivers (6 months, 69.0%; 6 years, 81.1%) rated their subjective stress as moderate or higher at both 6 months and 6 years. The patient–caregiver relationship level showed a considerable improvement from 6 months to 6 years after stroke onset (Z = −1.977, *p* = 0.048). In contrast, self-rated health and stress did not show any improvement at 6 years post-stroke. The proportion of caregivers living with patients remained the same for 6 years. All other characteristics, including care at home, care in hospital, use of social services, psychosocial stress, and quality of life, did not change over time.

### 3.3. Caregiver Burden and Changes over Time

**Figure 2:**
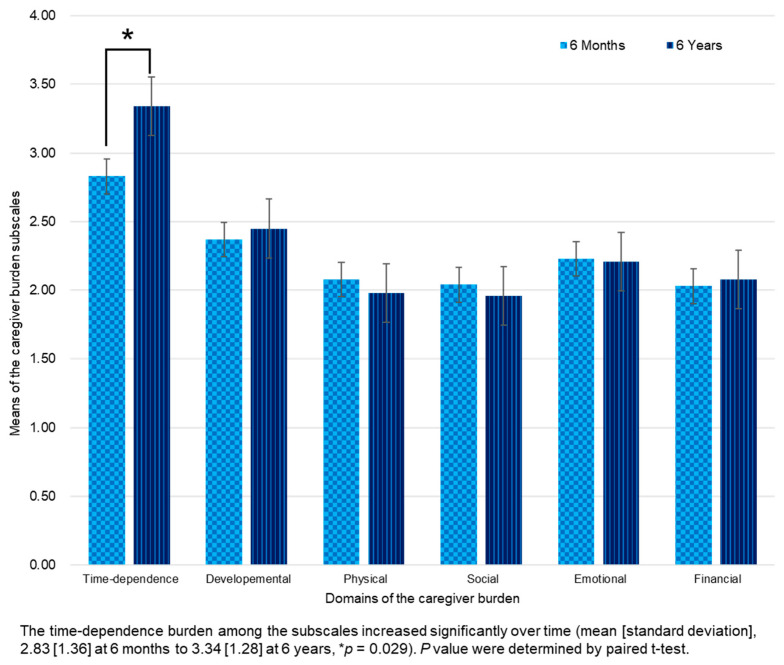
Change of caregiver burden subscale scores from 6 months to 6 years after stroke.

A high caregiver burden, which suggests a risk of burnout, was reported by 37.9% and 51.7% of caregivers at 6 months and 6 years post-stroke, respectively (Table 4 and Figure 2). Among the six domains of burden, time-dependence and developmental domain scores remained consistently the highest for up to 6 years. Both the caregiver burden total score and proportion of caregivers at risk of burnout did not decrease between 6 months and 6 years. The time-dependence burden was increased over time (mean [SD], 2.83 [1.36] to 3.34 [1.28], *p* = 0.029).

### 3.4. Associations and Predictors of High Caregiver Burden

Table 5 presents variables significantly associated with a high caregiver burden at 6 months and 6 years after stroke. At 6 months, the patients’ functional and emotional status (K-MMSE for cognitive function, FMA for motor function, FAC for mobility function, mRS for disability, K-MBI for activities of daily living, and EQ-5D for quality of life), the patients’ children (rather than spouses), the presence of alternative caregiver, and the caregivers’ relationship level, education level, self-rated health and stress, psychosocial stress (PWI-SF), and quality of life (EQ-5D) were related to the caregiver burden. Similarly, at 6 years, the patients’ functional and emotional status and the caregivers’ self-rated health and stress and psychosocial stress (PWI-SF) were factors associated with the caregiver burden.

**Figure 3:**
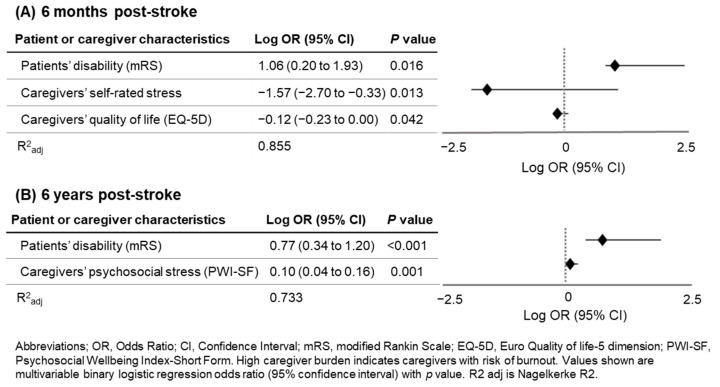
Forest plot and log OR of high caregiver burden at 6 months and 6 years after stroke.

Multivariable regression analysis with significant factors was performed to evaluate caregiver burden predictors at 6 months and 6 years (Table 6 and Figure 3). The following were identified as independent caregiver burden predictors at 6 months: patients’ disability (mRS) (odds ratio (OR) = 11.60; 95% confidence interval (CI) 1.58–85.08; *p* = 0.016), caregivers’ self-rated stress (OR = 0.03; 95% CI 0.00–0.47; *p* = 0.013), and caregivers’ quality of life (EQ-5D) (OR = 0.76; 95% CI 0.59–0.99; *p* = 0.042). At 6 years, only the patients’ disability (mRS) (OR = 5.88; 95% CI 2.19–15.82; *p* < 0.001) and caregivers’ psychosocial stress (PWI-SF) (OR = 1.26; 95% CI 1.10–1.44; *p* = 0.001) showed significance. At both time points, caregiver burden could be explained by more than 70% of the regression equation (85.5% at 6 months; 73.3% at 6 years).

## 4. Discussion

In the present study, we identified the patients’ disability and caregivers’ quality of life as burden predictors at 6 months, and the caregiver burden total score and proportion of caregivers at risk of burnout did not decrease between 6 months and 6 years. At 6 years, only the patients’ disability and caregivers’ psychosocial stress showed significance.

### 4.1. Predictors of Caregiver Burden

#### 4.1.1. Patient’s Disability and Psychosocial Stress

The patients’ disability, which could affect their activities of daily living, was identified as a caregiver burden predictor for up to 6 years post-stroke. Among the patients’ functions, functions related to mobility (FAC and mRS) rather than cognitive function (K-MMSE), motor function of the upper and lower extremities (FMA), and swallowing function (ASHA-NOMS) were more closely associated with caregiver burden. This finding reflects the important role of the patients’ mobility function in the actual caregiving process. A previous study of the KOSCO reported that factors related to the burden on caregivers of stroke survivors at 3 months after stroke onset were four or more comorbidities, neurological impairment at early onset, and dependence for activities of daily living, which were associated with a heavier burden of care [22]. Several studies also showed that dependency on activities of daily living and neurologic deficits among stroke survivors were strongly related to the burden on caregivers in not only the subacute stage but also the chronic stage [12,13,14,15,16,19,20,21,22,45,46].

The caregivers’ stress was the main caregiver burden predictor in both subacute and chronic phases. Consistent with our study, caregiver anxiety was the only significant predictor at 5 years post-stroke in a previous study [23]. A hospital-based study indicated that a factor responsible for major stress among caregivers was anxiety [47]. A recent systematic review suggested that anxiety and depression could increase caregiver burden [48].

#### 4.1.2. Caregivers’ Burnout in Long-Term Care

Among the six domains of caregiver burden, time-dependence and developmental domain scores remained consistently the highest for up to 6 years. In addition, the score of the time-dependence domain increased significantly over time for up to 6 years. The level of overall caregiver burden did not decrease over 6 years post-stroke, and the proportion of caregivers at risk of burnout also did not decrease; however, a gradual burden improvement associated with adaptation to new situations was observed in previous long-term follow-up studies [23,24]. Contrary to the method used in the studies mentioned above, the questionnaire in our study did not reflect the actual provided care hours or labor intensity [49,50]. In addition, the studies cited above were conducted in Europe, whereas this study investigated caregivers in an East Asian country (Korea). Therefore, although Asian caregivers may eventually adapt to a new situation over time, they cannot escape from the burden of long-term caregiving due to the influence of Confucianism, which promotes self-sacrifice for sick family members.

### 4.2. Strategies to Reduce Caregiver Burden

To reduce caregiver burden, emotional support services should be provided to caregivers with a high risk of caregiver burden. Providing caregivers with stress relief options can be vital for reducing the burden on stroke caregivers in all stages. Mental health professionals may play an important role in emotional support, stress management, and interventions for depression and anxiety [48]. Reducing the caregiving hours, especially in the initial months, may help reduce caregiver burden [46]. Furthermore, integrating technology into caregiver support systems, such as telehealth services and mobile applications, may help reduce caregiver burden [46]. A comprehensive program for a multiple-component, theory and evidence-based behavior change intervention could be an effective approach for reducing the burden on caregivers [51].

The use of social services was not correlated with caregiver burden, which is consistent with the results of previous studies showing that social service use was not associated with caregiver burden alleviation [14,52,53]. Only around 5% of caregivers used social services, which is consistent with a previous report [53]. Other studies found that Asian caregivers prefer informal assistance from family members over formal services, due to a lack of trust and limited information [54,55]. Therefore, there is a need to clarify the reasons for the low utilization of social services in South Korea.

### 4.3. Limitations

The present study has several limitations. A low proportion of caregivers completed the survey at 6 years post-stroke, preventing the generalization of the results. Additionally, there was selection bias, as caregivers who did not respond or whose patients had died were not included. When comparing the responder and non-responder groups, there was no difference in any of the caregiver-specific variables; however, most of the patient-specific factors showed significant differences. Particularly, the non-responder group had milder neurological symptoms and better functional status. Patients who can independently perform activities of daily living and do not require caregivers would tend not to complete the caregiver survey. Furthermore, this study is limited by its retrospective design and may have been influenced by unmeasured confounders.

## 5. Conclusions

Around half of the caregivers experienced a significant burden during the subacute phase of stroke, which lasted until the chronic phase of 6 years. The findings of this study suggest that consistent interventions, such as emotional support or counseling on stress relief strategies for caregivers of stroke survivors, may reduce caregiver burden. Further research is needed to establish specific strategies appropriate for Korean caregivers to alleviate their burden in caring for stroke patients.

## Figures and Tables

**Table 1:** Comparison of the included and excluded groups.

	Responders (*n* = 58)	Non-Responders (*n* = 834)	*p* Value ^a^
Patient characteristics ^b^			
Age, mean (SD), decades	7.45 (1.14)	6.93 (1.21)	0.001
Male gender, *n* (%)	30 (51.7)	482 (57.8)	0.366
Ischemic stroke, *n* (%)	48 (82.8)	652 (78.2)	0.412
Premorbid mRS, median (IQR)	0.00 (0.00–0.00)	0.00 (0.00–1.00)	0.014
Comorbidity, CCAS, mean (SD)	5.07 (1.68)	5.24 (1.53)	0.418
Comorbidity, WIC, mean (SD)	2.52 (0.76)	2.70 (0.85)	0.106
Initial NIHSS, mean (SD)	7.34 (9.10)	4.72 (6.45)	0.035
K-MMSE, mean (SD) ^c^	17.40 (10.02)	24.16 (7.01)	<0.001
FMA, mean (SD) ^c^	78.84 (30.26)	87.15 (25.16)	0.045
FAC, median (IQR) ^c^	4.00 (3.00–5.00)	5.00 (4.00–5.00)	0.002
ASHA-NOMS, median (IQR) ^c^	7.00 (7.00–7.00)	7.00 (7.00–7.00)	0.382
K-FAST, mean (SD) ^d^	16.45 (9.39)	21.56 (8.73)	<0.001
mRS, median (IQR) ^c^	2.00 (1.00–3.00)	1.00 (1.00–2.00)	<0.001
K-MBI, mean (SD) ^c^	80.52 (28.28)	88.76 (21.54)	0.033
GDS-SF, mean (SD) ^e^	6.97 (3.93)	5.53 (4.03)	0.029
EQ-5D, mean (SD)	52.81 (39.87)	72.20 (30.83)	<0.001
Caregiver characteristics			
Age, mean (SD), decades	6.02 (1.17)	5.97 (1.36)	0.786
Male gender, *n* (%)	20 (34.5)	290 (34.8)	0.964
Employment, *n* (%)	23 (39.7)	347 (41.6)	0.771
Education, *n* (%) ^f^			
Primary school and below	7 (12.1)	141 (16.9)	0.937
Middle school	9 (15.5)	113 (13.5)	
High school	23 (39.7)	279 (33.5)	
College and above	15 (25.9	267 (32.0)	
Relationship with patient, *n* (%)			
Spouse	28 (48.3)	486 (58.3)	0.577
Children	25 (43.1)	261 (31.3)	
Daughter-in-law	4 (6.9)	27 (3.2)	
Paid caregiver	0	6 (0.7)	
Grandchildren	0	4 (0.5)	
Parent	1 (1.7)	16 (1.9)	
Other	0	34 (4.1)	
Level of relationship, *n* (%)			
Very good	14 (24.1)	293 (35.1)	0.058
Good	30 (51.7)	394 (47.2)	
Average	12 (20.7)	134 (16.1)	
Bad	2 (3.4)	12 (1.4)	
Very bad	0	1 (0.1)	
Cohabitation with patient, *n* (%)	40 (69.0)	635 (76.1)	0.218
Care at home, *n* (%)	44 (75.9)	697 (83.6)	0.130
Consignment to long-term care institution, *n* (%)	4 (6.9)	42 (5.0)	0.536
Care in hospital, *n* (%)	7 (12.1)	75 (9.0)	0.433
Use of social services, *n* (%)	0	10 (1.2)	0.402
Presence of alternative caregiver, *n* (%)	26 (44.8)	280 (33.6)	0.081
Self-rated health, *n* (%)			
Very good	5 (8.6)	65 (7.8)	0.257
Good	15 (25.9)	284 (34.1)	
Average	22 (37.9)	332 (39.8)	
Bad	16 (27.6)	137 (16.4)	
Very bad	0	16 (1.9)	
Self-rated stress, *n* (%)			
Extremely	11 (19.0)	67 (8.0)	0.080
Very	13 (22.4)	182 (21.8)	
Moderately	16 (27.6)	278 (33.3)	
Slightly	8 (13.8)	161 (19.3)	
Not at all	10 (17.2)	146 (17.5)	
Knowledge of stroke, *n* (%)			
Motor symptoms	50 (86.2)	652 (78.2)	0.149
Speech symptoms	47 (81.0)	661 (79.3)	0.746
Visual symptoms	38 (65.5)	481 (57.7)	0.242
Ataxic symptoms	48 (82.8)	644 (77.2)	0.328
Acute stage treatment	44 (75.9)	579 (69.4)	0.302
Caregiver burden, mean (SD)	33.62 (15.10)	30.99 (13.93)	0.167
Psychosocial stress, PWI-SF, mean (SD)	17.57 (10.14)	18.03 (10.97)	0.756
Quality of life, EQ-5D, mean (SD) ^g^	89.86 (7.96)	90.96 (7.64)	0.292

Abbreviations: mRS, modified Rankin Scale; CCAS, Combined Condition and Age-related Score; WIC, Weighted Index of Comorbidity; NIHSS, National Institute of Health Stroke Scale; K-MMSE, Korean Mini Mental State Examination; FMA, Fugl-Meyer Assessment; FAC, Functional Ambulatory Category; ASHA-NOMS, American Speech-language Hearing Association National Outcome Measurement System Swallowing Scale; K-FAST, Korean Version of the Frenchay Aphasia Screening Test; K-MBI, Korean Modified Barthel Index; GDS-SF, Geriatric Depression Scale-Short Form; EQ-5D, Euro Quality of Life-5 Dimension; PWI-SF, Psychosocial Wellbeing Index-Short Form. ^a^ *p* values were determined by *t*-test, $\chi^{2}$ test, and linear-by-linear association. ^b^ Patients’ functional status at 6 months post-stroke was compared. ^c^ Missing data for K-MMSE, FMA, FAC, ASHA-NOMS, mRS, and K-MBI among non-responders (2 [0.24%]). ^d^ Missing data for K-FAST among responders (9 [15.5%]) and non-responders (22 [2.64%]). ^e^ Missing data for GDS-SF among responders (19 [32.8%]) and non-responders (100 [12.0%]). ^f^ Missing data for education among responders (4 [6.90%]) and non-responders (34 [4.08%]). ^g^ EQ-5D total score was multiplied by 100.

**Table 2:** Patient characteristics at 6 months and 6 years after stroke.

	Score Range (Bad to Good)	6 Months	6 Years	*p* Value ^b^
Age, mean (SD), decades		7.45 (1.14)		
Male gender, *n* (%)		30 (51.7)		
Ischemic stroke, *n* (%)		48 (82.8)		
Premorbid mRS, median (IQR)	6–0	0.00 (0.00–0.00)		
Comorbidity, CCAS, mean (SD)	16–1	5.07 (1.68)		
Comorbidity, WIC, mean (SD)	13–1	2.52 (0.76)		
Initial NIHSS, mean (SD)	42–0	7.34 (9.10)		
K-MMSE, mean (SD)	0–30	17.40 (10.02)	13.62 (11.91)	0.002
FMA, mean (SD)	0–100	78.84 (30.26)	77.78 (29.73)	0.731
FAC, median (IQR)	0–5	4.00 (3.00–5.00)	4.00 (2.00–5.00)	0.208
ASHA-NOMS, median (IQR)	1–7	7.00 (7.00–7.00)	7.00 (6.00–7.00)	0.083
K-FAST, mean (SD) ^a^	0–30	16.45 (9.39)	15.70 (10.84)	0.909
mRS, median (IQR)	6–0	2.00 (1.00–3.00)	2.50 (1.00–4.00)	0.183
K-MBI, mean (SD)	0–100	80.52 (28.28)	72.00 (30.94)	0.007
GDS-SF, mean (SD) ^a^	15–0	6.97 (3.93)	2.36 (3.71)	<0.001
EQ-5D, mean (SD) ^c^	−1.7–100	52.81 (39.87)	38.24 (43.43)	0.023

Abbreviations: mRS, modified Rankin Scale; CCAS, Combined Condition and Age-related Score; WIC, Weighted Index of Comorbidity; NIHSS, National Institute of Health Stroke Scale; K-MMSE, Korean Mini Mental State Examination; FMA, Fugl-Meyer Assessment; FAC, Functional Ambulatory Category; ASHA-NOMS, American Speech-language Hearing Association National Outcome Measurement System Swallowing Scale; K-FAST, Korean Version of the Frenchay Aphasia Screening Test; K-MBI, Korean Modified Barthel Index; GDS-SF, Geriatric Depression Scale-Short Form; EQ-5D, Euro Quality of Life-5 Dimension. ^a^ Variables with a missing ratio of 10% or more were excluded from subsequent data analysis. Missing data for K-FAST at 6 months (9 [15.5%]) and 6 years (18 [31.0%]) and for GDS-SF at 6 months (19 [32.8%]). ^b^ *p* values were determined by paired *t*-test and Wilcoxon test. ^c^ EQ-5D total score was multiplied by 100.

**Table 3:** Caregiver characteristics at 6 months and 6 years after stroke.

	6 Months	6 Years	*p* Value ^b^
Age, mean (SD), decades	6.02 (1.17)	6.64 (1.21)	<0.001
Male gender, *n* (%)	20 (34.5)	17 (29.3)	0.549
Employment, *n* (%)	23 (39.7)	23 (39.7)	1.000
Education ^a^, *n* (%)			
Primary school and below	7 (12.1)	8 (13.8)	0.211
Middle school	9 (15.5)	12 (20.7)	
High school	23 (39.7)	21 (36.2)	
College and above	15 (25.9)	14 (24.1)	
Relationship with patient, *n* (%)			
Spouse	28 (48.3)	27 (46.6)	0.417
Children	25 (43.1)	26 (44.8)	
Daughter-in-law	4 (6.9)	2 (3.4)	
Paid caregiver	0	2 (3.4)	
Grandchildren	0	0	
Parent	1 (1.7)	1 (1.7)	
Other	0	0	
Level of relationship, *n* (%)			
Very good	14 (24.1)	24 (41.4)	0.048
Good	30 (51.7)	22 (37.9)	
Average	12 (20.7)	12 (20.7)	
Bad	2 (3.4)	0	
Very bad	0	0	
Cohabitation with patient, *n* (%)	40 (69.0)	37 (63.8)	0.508
Care at home, *n* (%)	44 (75.9)	44 (75.9)	1.000
Consignment to long-term care institution, *n* (%)	4 (6.9)	8 (13.8)	0.289
Care in hospital, *n* (%)	7 (12.1)	2 (3.4)	0.125
Use of social services, *n* (%)	0	3 (5.2)	0.250
Presence of alternative caregiver, *n* (%)	26 (44.8)	22 (37.9)	0.481
Self-rated health, *n* (%)			
Very good	5 (8.6)	4 (6.9)	0.537
Good	15 (25.9)	15 (25.9)	
Average	22 (37.9)	31 (53.4)	
Bad	16 (27.6)	8 (13.8)	
Very bad	0	0	
Self-rated stress, *n* (%)			
Extremely	11 (19.0)	5 (8.6)	0.418
Very	13 (22.4)	19 (32.8)	
Moderately	16 (27.6)	23 (39.7)	
Slightly	8 (13.8)	6 (10.3)	
Not at all	10 (17.2)	5 (8.6)	
Knowledge of stroke, *n* (%)			
Motor symptoms	50 (86.2)	44 (75.9)	0.263
Speech symptoms	47 (81.0)	45 (77.6)	0.824
Visual symptoms	38 (65.5)	34 (58.6)	0.541
Ataxic symptoms	48 (82.8)	46 (79.3)	0.832
Acute stage treatment	44 (75.9)	38 (65.5)	0.286
Psychosocial stress, PWI-SF, mean (SD)	17.57 (10.14)	16.52 (11.47)	0.516
Quality of life, EQ-5D, mean (SD) ^c^	89.86 (7.96)	92.16 (5.92)	0.050

Abbreviations: PWI-SF, Psychosocial Wellbeing Index-Short Form; EQ-5D, Euro Quality of Life-5 Dimension. ^a^ Missing data at 6 months (4 [6.9%]) and 6 years (3 [5.2%]). ^b^ *p* values were determined by paired *t*-test, McNemar’s test, and Wilcoxon test. ^c^ EQ-5D total score was multiplied by 100.

**Table 4:** Caregiver burden at 6 months and 6 years after stroke ^a^.

	6 Months	6 Years	*p* Value ^b^
Caregiver burden total score, mean (SD)	33.62 (15.10)	34.38 (13.62)	0.718
Time-dependence domain, mean (SD)	2.83 (1.36)	3.34 (1.28)	0.029
Developmental domain, mean (SD)	2.37 (1.28)	2.45 (1.17)	0.640
Physical domain, mean (SD)	2.08 (1.16)	1.98 (1.07)	0.595
Social domain, mean (SD)	2.04 (0.91)	1.96 (0.90)	0.542
Emotional domain, mean (SD)	2.23 (1.16)	2.21 (1.07)	0.891
Financial domain, mean (SD)	2.03 (0.98)	2.08 (0.90)	0.753
High caregiver burden, *n* (%) ^c^	22 (37.9)	30 (51.7)	0.077

**^a^** The means of six subscales were calculated for comparison according to the burden domain, with a score ranging from 1 to 5. ^b^ *p* values were determined by paired *t*-test or McNemar’s test. ^c^ A high caregiver burden indicates a risk of burnout.

**Table 5:** Association of patient and caregiver characteristics with high caregiver burden (risk of burnout) at 6 months and 6 years after stroke.

	6 Months		6 Years	
	OR (95% CI)	*p* Value	OR (95% CI)	*p* Value
**Patient characteristics ^a^**				
Initial NIHSS	1.19 (1.06–1.33)	0.002		
Cognitive function (K-MMSE)	0.91 (0.86–0.97)	0.002		
Motor function (FMA)	0.96 (0.94–0.98)	0.001	0.95 (0.92–0.98)	0.003
Mobility function (FAC)	0.38 (0.23–0.63)	<0.001	0.44 (0.28–0.70)	<0.001
Swallowing function (ASHA-NOMS)			0.41 (0.17–0.99)	0.048
Disability (mRS)	3.41 (1.83–6.35)	<0.001	2.93 (1.70–5.04)	<0.001
Activities of daily living (K-MBI)	0.94 (0.91–0.98)	0.002	0.96 (0.93–0.98)	<0.001
Quality of life (EQ-5D)	0.97 (0.95–0.98)	<0.001	0.98 (0.97–0.99)	0.004
**Caregiver characteristics ^a^**				
Education	0.37 (0.18–0.73)	0.004		
Patients’ children (reference = spouse) ^b^	0.16 (0.05–0.56)	0.004		
Level of relationship	2.29 (1.06–4.93)	0.035		
Presence of alternative caregiver	0.21 (0.06–0.70)	0.011		
Self-rated health	2.54 (1.26–5.13)	0.009	2.32 (1.09–4.96)	0.029
Self-rated stress	0.19 (0.08–0.44)	<0.001	0.30 (0.14–0.63)	0.002
Psychosocial stress (PWI-SF)	1.19 (1.09–1.31)	<0.001	1.13 (1.05–1.21)	<0.001
Quality of life (EQ-5D)	0.88 (0.81–0.96)	0.005		

Abbreviations: OR, odds ratio; CI, confidence interval; NIHSS, National Institute of Health Stroke Scale; K-MMSE, Korean Mini Mental State Examination; FMA, Fugl-Meyer Assessment; FAC, Functional Ambulatory Category; ASHA-NOMS, American Speech-language Hearing Association National Outcome Measurement System Swallowing Scale; mRS, modified Rankin Scale; K-MBI, Korean Modified Barthel Index; EQ-5D, Euro Quality of Life-5 Dimension; PWI-SF, Psychosocial Wellbeing Index-Short Form. ^a^ *p* values were determined by *t*-test or $\chi^{2}$ test. ^b^ *p* values were determined by univariable logistic regression analysis.

**Table 6:** Predictors of high caregiver burden at 6 months and 6 years after stroke.

	6 Months		6 Years	
	OR (95% CI) ^a^	*p* Value	OR (95% CI) ^a^	*p* Value
**Patient characteristics**				
Disability (mRS)	11.60 (1.58–85.08)	0.016	5.88 (2.19–15.82)	<0.001
**Caregiver characteristics**				
Self-rated stress	0.03 (0.00–0.47)	0.016		
Psychosocial stress (PWI-SF)			1.26 (1.10–1.44)	0.001
Quality of life (EQ-5D)	0.76 (0.59–0.99)	0.042		
**R^2^_adj_ ^b^**	0.855		0.733	

Abbreviations: OR, odds ratio; CI, confidence interval; mRS, modified Rankin Scale; PWI-SF, Psychosocial Wellbeing Index-Short Form; EQ-5D, Euro Quality of Life-5 Dimension. ^a^ Values shown are the multivariable binary logistic regression odds ratio (95% confidence interval). ^b^ Nagelkerke R^2^.

## Data Availability

The datasets generated during and/or analyzed during the current study are available from the corresponding author on reasonable request.
